# Hepatitis A outbreak among men who have sex with men, Shinjuku, Japan, 2018

**DOI:** 10.5365/wpsar.2025.16.1.1088

**Published:** 2025-02-25

**Authors:** Mariya Itaki, Masayuki Endo, Hiroyuki Asakura, Mami Nagashima, Yoshiko Somura, Aki Takahashi, Aya Kayebeta, Ikumi Takahashi, Yuichiro Yahata

**Affiliations:** a Department of Health, Shinjuku City Office, Tokyo, Japan.; b Department of Microbiology, Tokyo Metropolitan Institute of Public Health, Tokyo, Japan.; c Infectious Diseases Surveillance Center, National Institute of Infectious Diseases, Tokyo, Japan.

## Abstract

**Objective:**

In 2018, the Shinjuku City Department of Health detected excess cases of hepatitis A virus (HAV) infection. The objectives of this investigation were to characterize the outbreak, identify transmission routes among inpatient cases and make recommendations to control and prevent HAV infection among men who have sex with men.

**Methods:**

Information about cases of HAV infection was collected from the National Epidemiological Surveillance for Infectious Diseases system and inpatient interviews conducted by public health nurses in 2018.

**Results:**

There were 131 HAV cases in 2018. Of these, 98% (129/131) were male, of whom 81% (105/129) were men who have sex with men. Hospitalization was required for 40 cases (31%). The age groups with the highest proportion of cases were 30–39 and 40–49 years (each 34%; 44/131). Two cases (2%) had received the second dose of the HAV vaccine, but only 10 days before symptom onset; all others had received no doses. The sequence type subgroup 13, an RIVM-HAV-16–090-like strain, was seen in 51 cases (39%). Of the 40 hospitalized cases, 21 (53%) participated in an interview conducted using a semistructured questionnaire. Altogether, of 21 cases, 12 (57%) had coinfection with HIV, 13 (62%) had casual sexual contact within the preceding 2 months and 10 (48%) had used social networking services (SNS) to find a sexual partner.

**Discussion:**

In Shinjuku, this outbreak almost exclusively affected the population of men who have sex with men. The detected outbreak strain has previously been reported in outbreaks among men who have sex with men in China, Taiwan (China) and Europe. For HAV prevention, the most important measures are raising awareness of the risk of HAV as a sexually transmitted infection via SNS and promoting immunization at the appropriate time.

Hepatitis A virus (HAV) is transmitted person-to-person through the faecal–oral route or by ingestion of contaminated food or water. (*1*, *2*) In countries where HAV is not endemic, the onset of illness among adults is usually abrupt, comprising fever, malaise, anorexia, nausea and abdominal discomfort, followed within a few days by jaundice. Since June 2015, outbreaks of HAV infection with particular strains have emerged among men who have sex with men in China, Taiwan (China) and in European countries. (*2*-*4*) Thus, HAV infection is a major re-emerging infectious disease among populations of men who have sex with men in developed countries. The strain mainly implicated among these groups and patients with HIV infection or other sexually transmitted infections (STIs) is TA-15 (RIVM-HAV16–090). (*1*)

Shinjuku is one of the special wards in Tokyo that has its own public health administration and local public health centre (PHC), as authorized in the Community Health Act. (*5*) It is host to the Tokyo Metropolitan Government Building and the head offices of many major corporations, and had a population of around 347 000, as of the end of 2018. (*6*) Shinjuku is known for its gay quarter, Shinjuku 2-chome, with more than 400 commercial recreational facilities that cater to the LGBTQ+ (lesbian, gay, bisexual, transgender, intersex, queer/questioning, asexual and others) community. (*7*)

HAV infection is classified as a category IV notifiable disease in Japan, in accordance with the Act on the Prevention of Infectious Diseases and Medical Care for Patients with Infectious Diseases. (*8*) The annual number of HAV infections nationwide ranged from around 100 to 350 cases in 2004–2014. (*9*) An excess number of cases of HAV infection was reported in 2018, with 177 cases diagnosed nationwide in the first 15 weeks of the year. (*10*) As of week 7 of the epidemic (18 February 2018), Shinjuku’s PHC had recorded 10 HAV cases, exceeding the threshold for declaring an outbreak, with some severe cases requiring inpatient care. The PHC initiated an outbreak investigation and established control measures. Some reports have described HAV outbreaks since 2018. (*7*, *11*-*13*) However, no study in Japan has investigated the prevention of HAV infection among men who have sex with men and who engage in high-risk sexual behaviours. The objectives of this investigation were to characterize the outbreak, to identify transmission routes among inpatient cases, and to make recommendations for the control and prevention of HAV infection among men who have sex with men.

## Methods

Two types of data analysis were conducted for this outbreak investigation: the first was a descriptive cross-sectional study that involved analysing cases of HAV infection recorded in the National Epidemiological Surveillance for Infectious Diseases (NESID) system, and the second involved reviewing the interviews conducted with inpatients in 2018. The interview data were descriptively analysed.

Data were extracted from NESID about individuals diagnosed with HAV at medical institutions in Shinjuku from 1 January to 31 December 2018; the characteristics analysed included age, sex, transmission route, molecular analysis of HAV strain, and other recorded data. Descriptive statistics were calculated using SAS v. 9.4 (SAS Institute Inc., Cary, NC, USA).

Molecular typing – including reverse transcription polymerase chain reaction (RT–PCR), sequence analysis and phylogenetic tree analysis – was conducted by the Tokyo Metropolitan Institute of Public Health, as previously reported by Ishii et al. (*14*)

Interviews were conducted in January–December 2018 with 21 hospitalized patients who had severe HAV infection, with the aim of learning how to prevent transmission and severe complications of infection in the community of men who have sex with men. The interviews were conducted by public health nurses from the Shinjuku PHC using a semistructured questionnaire. The questionnaire had 13 items, with questions about transmission route and infection prevention measures; it included questions about lifestyle factors, types of sexual partners, use of social networking services (SNS) to find sexual partners, whether the respondent visited gay cruising spots, the number of casual sexual contacts, condom use, knowledge of the HAV epidemic, whether the respondent was employed in food handling and the respondent’s HAV vaccination status. The interviews were conducted as part of the legal requirements for outbreak prevention and response activities, and informed consent for interviews was obtained from patients and their doctors. The participants had the right to decline to answer any interview item.

## Results

### Surveillance data from Shinjuku

**Fig. 1:**
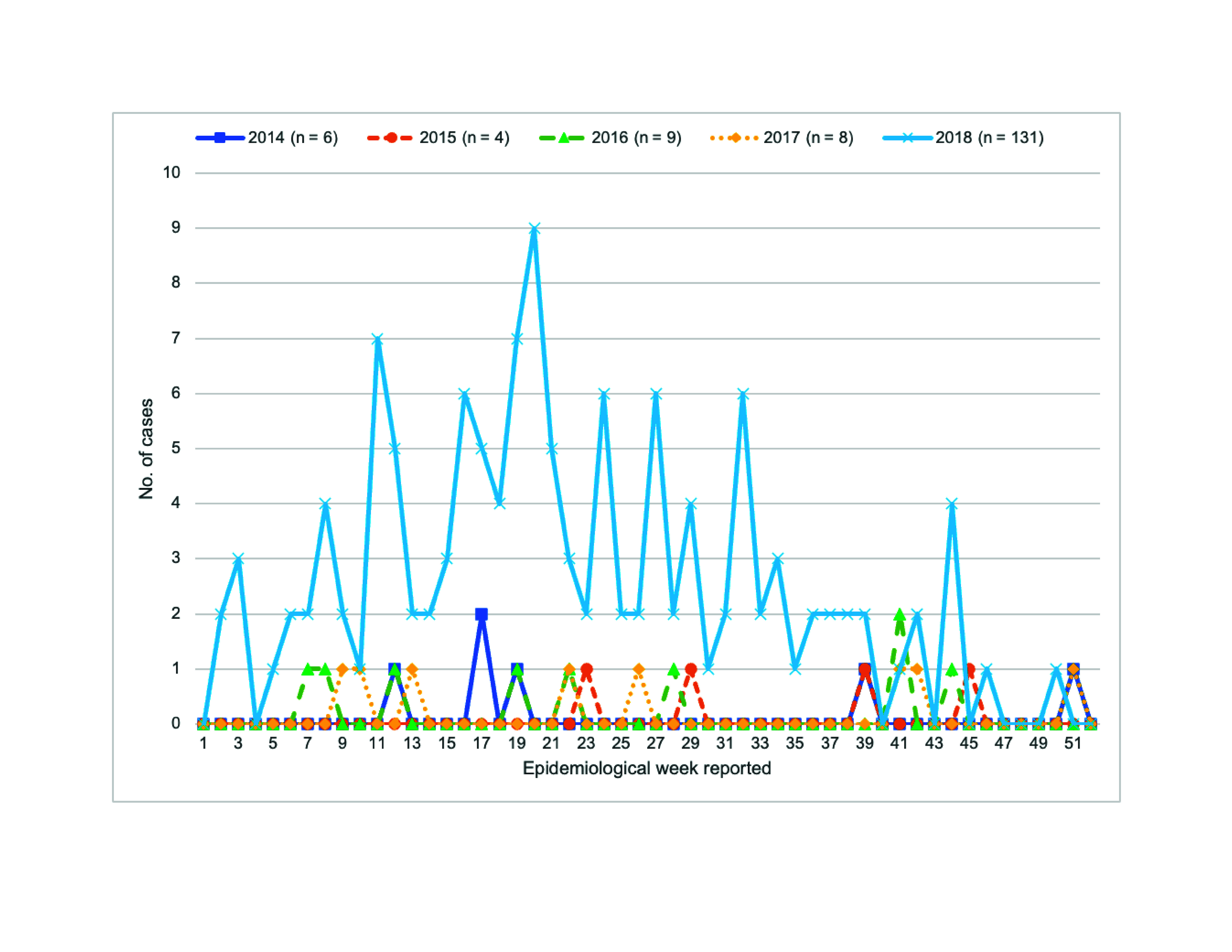
Number of weekly confirmed cases of acute hepatitis A infection by week and year of diagnosis, Shinjuku, Japan, 2014–2018 (N = 158)

The number of HAV cases in 2018 was 131; males comprised 98% (129/131; **Table 1**). For comparison, Shinjuku reported fewer than 10 cases of HAV infection annually from 2014 to 2017, totalling only 27 cases during that period (**Fig. 1**). Of the 131 cases, 126 (96%) were reported from a hospital designated by the government to provide medical services to people living with HIV (i.e. a designated HIV hospital) and were initially detected during a routine health check. The suspected source of infection was same-sex sexual contact in 81% of male cases (105/129) (**Table 1**) but was oral ingestion in the two female cases. In 2018, the most common age groups infected with HAV were those aged 30–39 years and 40–49 years (each 34%; 44/131; **Table 1**). The most common initial symptoms of HAV infection were malaise (113 cases; 86%), liver dysfunction  (109 cases; 83%) and fever (93 cases; 71%). HAV infection was diagnosed based on immunoglobulin testing, IgM (130 cases; 99%), IgG (1; 1%) or RT–PCR (59; 45%), or a combination of these. The high rate of positivity for the IgM test included an asymptomatic case detected by a blood test. Only 2 cases (2%) had received the second dose of the HAV vaccine.

[**insert** Figure 1]

**Table 1:** Characteristics of hepatitis A cases by sex, Shinjuku, Japan, 2018 (*n* = 131)

Characteristic	Female (*n* = 2)^a^	Male (*n* = 129)^a^	Total (*n* = 131)^a^
**Age group (years)**			
**10–19**	**0 (0)**	**2 (2)**	**2 (2)**
**20–29**	**0 (0)**	**29 (22)**	**29 (22)**
**30–39**	**0 (0)**	**44 (34)**	**44 (34)**
**40–49**	**1 (50)**	**43 (33)**	**44 (34)**
**50–59**	**1 (50)**	**10 (8)**	**11 (8)**
**60–69**	**0 (0)**	**1 (1)**	**1 (1)**
**Symptom**			
**Malaise**	**1 (50)**	**112 (87)**	**113 (86)**
**Fever**	**2 (100)**	**91 (71)**	**93 (71)**
**Lack of appetite**	**1 (50)**	**79 (61)**	**80 (61)**
**Jaundice**	**0 (0)**	**82 (64)**	**82 (63)**
**Hepatomegaly**	**0 (0)**	**26 (20)**	**26 (20)**
**Liver dysfunction**	**1 (50)**	**108 (84)**	**109 (83)**
**Upper abdominal pain**	**0 (0)**	**1 (1)**	**1 (1)**
**Diarrhoea**	**0 (0)**	**1 (1)**	**1 (1)**
**Pale stool**	**0 (0)**	**1 (1)**	**1 (1)**
**Dark urine**	**0 (0)**	**1 (1)**	**1 (1)**
**Joint pain**	**0 (0)**	**1 (1)**	**1 (1)**
**Asymptomatic**	**0 (0)**	**1 (1)**	**1 (1)**
**Method of testing**			
**IgM**	**2 (100)**	**128 (99)**	**130 (99)**
**IgG/paired**	**0 (0)**	**1 (1)**	**1 (1)**
**RT–PCR**	**2 (100)**	**57 (44)**	**59 (45)**
**Immunization (at least first dose)**			
**Yes**	**0 (0)**	**2 (2)**	**2 (2)**
**No**	**1 (50)**	**52 (40)**	**53 (40)**
**Unknown**	**1 (50)**	**69 (53)**	**70 (53)**
**Not recorded**	**0 (0)**	**5 (4)**	**5 (4)**
**Reported medical institution**			
**Designated HIV hospital**	**2 (100)**	**124 (96)**	**126 (96)**
**Other**	**0 (0)**	**5 (4)**	**5 (4)**
**Strain**			
**Subgroup A**	**1 (50)**	**0 (0)**	**1 (1)**
**Subgroup B**	**1 (50)**	**50 (39)**	**51 (39)**
**Unknown/not recorded**	**0 (0)**	**79 (61)**	**79 (60)**
**Travelled abroad within 30 days before onset**	**0 (0)**	**0 (0)**	**0 (0)**
**Hospitalized**	**0 (0)**	**40 (31)**	**40 (31)**
**Suspected source of infection**			
**Same-sex sexual contact**	**0 (0)**	**105 (81)**	**105 (80)**
**Oral ingestion**	**2 (100)**	**10 (8)**	**12 (9)**
**Oral ingestion and same-sex sexual contact**	**0 (0)**	**5 (4)**	**5 (4)**
**Other**	**0 (0)**	**9 (7)**	**9 (7)**

IgG: immunoglobulin G; IgM: immunoglobulin M; RT–PCR: reverse transcription polymerase chain reaction.

^a^ Values are number (%).

Altogether, 40 cases (31%) were hospitalized with severe illness. The reporting medical institution was a designated HIV hospital for 126 cases (96%; 2 females and 124 males). The 2 female cases were not infected with HIV. Only 52/131 samples (40%) were sequenced for molecular typing. HAV subgenotype IA/subgroup 13 (S13), an RIVM-HAV-16–090-like strain, was identified in 51 samples (98%). The only hospitalized female case also had S13, but the suspected source of infection was food. S13 strains were registered in the GenBank database, with accession numbers shown in **Supplementary Table 1**. No cases had travelled abroad within 30 days before symptom onset.

### Interviews with hospitalized cases

Semistructured interviews were conducted with 22 of the 40 cases hospitalized with severe illness (55%), comprising 21 males and 1 female (**Table 2**). Among the 21 male cases, the most common age group was 30–39 years (7 cases; 33%) followed by 40–49 years (6 cases; 29%).

**Table 2:** Transmission route and risk factors identified in interviews with cases hospitalized with severe hepatitis A infection, by age group, Shinjuku, Japan, 2018 (*n* = 21)

Transmission route or risk factor	Age group^a^					Total (*n* = 21)^a^
	10–19 (*n* = 1)	20–29 (*n* = 5)	30–39 (*n* = 7)	40–49 (*n* = 6)	50–59 (*n* = 2)	
**Route**						
**Oral (food)**	**1 (100)**	**1 (20)**	**1 (14)**	**1 (17)**	**0**	**4 (19)**
**Same-sex sexual contact**	**0**	**4 (80)**	**6 (86)**	**5 (83)**	**2 (100)**	**17 (81)**
**HIV coinfection**						
**No**	**1 (100)**	**3 (60)**	**3 (43)**	**1 (17)**	**0**	**8 (38)**
**Yes**	**0**	**1 (20)**	**4 (57)**	**5 (83)**	**2 (100)**	**12 (57)**
**Not answered**	**0**	**1 (20)**	**0**	**0**	**0**	**1 (5)**
**Unspecified number of sexual contacts**						
**No**	**1 (100)**	**2 (40)**	**2 (29)**	**1 (17)**	**0**	**6 (29)**
**Yes**	**0**	**2 (40)**	**5 (71)**	**4 (67)**	**2 (100)**	**13 (62)**
**Not answered**	**0**	**1 (20)**	**0**	**1 (17)**	**0**	**2 (10)**
**Usual partner**						
**No**	**1 (100)**	**1 (20)**	**3 (43)**	**3 (50)**	**0**	**8 (38)**
**Yes**	**0**	**3 (60)**	**3 (43)**	**3 (50)**	**2 (100)**	**11 (52)**
**Not answered**	**0**	**1 (20)**	**1 (14)**	**0**	**0**	**2 (10)**
**Uses SNS to find sexual contacts**						
**No**	**1 (100)**	**0**	**2 (29)**	**2 (33)**	**1 (50)**	**6 (29)**
**Yes**	**0**	**3 (60)**	**4 (57)**	**2 (33)**	**1 (50)**	**10 (48)**
**Not answered**	**0**	**2 (40)**	**1 (14)**	**2 (33)**	**0**	**5 (24)**
**Gay cruising spot use**						
**No**	**1 (100)**	**3 (60)**	**4 (57)**	**1 (17)**	**0**	**9 (43)**
**Yes**	**0**	**0**	**2 (29)**	**4 (67)**	**2 (100)**	**8 (38)**
**Not answered**	**0**	**2 (40)**	**1 (14)**	**1 (17)**	**0**	**4 (19)**
**Condom use**						
**No**	**0**	**2 (40)**	**3 (43)**	**1 (17)**	**1 (50)**	**7 (33)**
**Yes**	**0**	**2 (40)**	**2 (29)**	**3 (50)**	**1 (50)**	**8 (38)**
**Not answered**	**1 (100)**	**1 (20)**	**2 (29)**	**2 (33)**	**0**	**6 (29)**
**Sexual contact with HAV-positive individual**						
**No**	**0**	**1 (20)**	**0**	**1 (17)**	**0**	**2 (10)**
**Yes**	**0**	**1 (20)**	**2 (29)**	**1 (17)**	**0**	**4 (19)**
**Not answered**	**1 (100)**	**3 (60)**	**5 (71)**	**4 (67)**	**2 (100)**	**15 (71)**
**Sexual contact with HIV-positive individual**						
**No**	**0**	**2 (40)**	**0**	**0**	**0**	**2 (10)**
**Yes**	**0**	**1 (20)**	**1 (14)**	**2 (33)**	**0**	**4 (19)**
**Not answered**	**1 (100)**	**2 (40)**	**6 (86)**	**4 (67)**	**2 (100)**	**15 (71)**
**Immunization**						
**No**	**0**	**4 (80)**	**7 (100)**	**4 (67)**	**2 (100)**	**17 (81)**
**Yes**	**0**	**0**	**0**	**2 (33)**	**0**	**2 (10)**
**Not answered**	**1 (100)**	**1 (20)**	**0**	**0**	**0**	**2 (10)**
**Aware of epidemic**						
**No**	**0**	**2 (40)**	**5 (71)**	**3 (50)**	**1 (50)**	**11 (52)**
**Yes**	**0**	**2 (40)**	**2 (29)**	**3 (50)**	**1 (50)**	**8 (38)**
**Not answered**	**1 (100)**	**1 (20)**	**0**	**0**	**0**	**2 (10)**
**Food handler**						
**No**	**1 (100)**	**3 (60)**	**5 (71)**	**6 (100)**	**2 (100)**	**17 (81)**
**Yes**	**0**	**2 (40)**	**2 (29)**	**0**	**0**	**4 (19)**
**Lives with a housemate**						
**No**	**0**	**3 (60)**	**3 (43)**	**3 (50)**	**1 (50)**	**10 (48)**
**Yes**	**1 (100)**	**2 (40)**	**4 (57)**	**3 (50)**	**1 (50)**	**11 (52)**

HAV: hepatitis A virus; SNS: social networking services.

^a^ Values are number (%).

For the transmission route in the 21 cases, same-sex sexual contact was suspected in 17 cases (81%). The numbers of cases with a specific risk factor were: 13 cases (62%) who had sexual contact with an unspecified number of persons within the preceding 2 months; 12 cases (57%) who were coinfected with HIV; 10 cases (48%) who found sexual partners using SNS; and 8 cases (38%) who had visited gay cruising spots. Two cases (10%) had received the HAV vaccine, 8 cases (38%) were aware of the current HAV epidemic and 4 cases (19%) were employed in food handling. For the 2 vaccinated cases, the HAV vaccine had been administered within 10 days before symptom onset.

Stratified by age group, around 60% among those in their 20s and 30s found sexual partners using SNS. Additionally, 6 of the 8 cases who visited gay cruising spots were in their 40s and 50s.

### Outbreak control measures

After the outbreak was detected, a few gay community voluntary support groups, the Tokyo Metropolitan Government and Shinjuku City Government discussed strategies for preventing HAV infection in the community of men who have sex with men. Some physicians at collaborating hospitals that offer treatment for HIV and AIDS in Tokyo recommended HAV vaccination for patients and their partners who were part of the population. Support groups were informed about the HAV epidemic through SNS and disseminated information about HAV infection in collaboration with the Tokyo Metropolitan Government and the PHCs of the 23 special wards of Tokyo, including Shinjuku PHC.

## Discussion

The data analysed indicated that the number of HAV infections rapidly increased in January 2018 in Shinjuku. In comparison with past surveillance data, in 2018 the most common transmission route among male cases was same-sex sexual contact (81%). This was most common among those in their 30s and 40s. Among the 21 male cases hospitalized with severe illness who consented to be interviewed, the most common risk factor for cases in their 30s and 40s was the use of SNS to find sexual partners. Only two of the cases had received any doses of the HAV vaccine. Genetic analysis identified the dominant virus strain as sequence type S13, an RIVM-HAV61–090-like strain. (*15*, *16*)

### Main affected population

This 2018 outbreak constituted the highest number of HAV infections recorded in Japan since 2014 (at which time 433 cases were reported nationwide), (*9*, *17*) and the outbreak was primarily confined to Shinjuku and the population of men who have sex with men. The rate of transmission via same-sex sexual contact among male cases has gradually been increasing since 2016. (*17*)

### Risk factors

Our results showed that those infected with HAV tended to have multiple casual partners, and half had coinfection with HIV. About half of the recorded cases found sexual partners using SNS, such as X.com, Facebook and Instagram, and about 60% of those in their 20s and 30s used these sites, which was a higher proportion than in other age groups. These age groups use SNS frequently and have access to more mobile communication tools. (*18*) The use of SNS to find sexual partners is a high-risk behaviour consistent with a previous HAV outbreak among this population. (*3*) Using SNS and meeting partners online were also associated with HIV-positive status and having an STI. (*19*, *20*) Thus, this population should be aware of the risk of HAV infection associated with these behaviours.

### Preventing HAV infection in this population

Around 60% of cases in this study were HIV-positive, which is consistent with previous reports. (*21*) HIV positivity among men who have sex with men is associated with a high risk of HAV infection, as is frequent oral–anal sexual contact (*22*) and having multiple sexual partners. (*21*) Moreover, according to Nishijima et al., (*23*) a hospital offering treatment for HIV and AIDS reported that around 90% of patients with HIV or AIDS in metropolitan areas of Japan were men who have sex with men. In the 2018 outbreak, most cases of HAV infection were reported by these HIV/AIDS hospitals, and cases of HAV infection were initially detected during routine health checks (data not shown) performed at the hospital in Shinjuku. In contrast, about 40% of HAV infections were reported by a non-HIV/AIDS hospital later in the outbreak. After the HAV outbreak was detected, voluntary support groups and the local government discussed how best to disseminate information about the outbreak and institute measures to control it, and the voluntary support groups subsequently disseminated information about the HAV outbreak among the affected population. (*24*) Communicating to the affected population that approximately half of the cases were not coinfected with HIV or AIDS might have generally increased awareness of the HAV outbreak in the population. SNS have been reported to be an important tool for communicating about infectious disease prevention measures and increasing the uptake of effective sexual health behaviours to reduce the risk of disease transmission. (*25*) To improve health behaviours to prevent HAV infection and to promote immunization, health-care providers and voluntary support groups should widely disseminate appropriate information via SNS.

### Outbreak strain

The dominant strain of HAV in this outbreak was sequence type S13, which is an RIVM-HAV16–090-like strain. (*15*) This sequence type was also identified in outbreaks among men who have sex with men in China, Taiwan (China) from 2015 to 2017, and in England and Germany in 2016 and 2017. (*2*-*4*) The RIVM-HAV16–090-like strain had not been reported in Japan before 2016. The strain has circulated among the population of men who have sex with men worldwide, and therefore the outbreak source was possibly importation of the RIVM-HAV16–090-like strain from epidemics in other countries, with subsequent transmission to the Japanese population of men who have sex with men in Shinjuku. Our data showed that in 2018 in Shinjuku, 81% of cases were men who have sex with men. Among those infected with the RIVM-HAV-16–090-like strain, the proportion of men who have sex with men was the same as in previous reports from European countries. For this population, sexual behaviour might facilitate transmission through close contact with someone who is infected. (*26*-*28*) In Japan, the RIVM-HAV-16–090-like strain was first reported in 2016, which also suggests the possibility that the strain was imported from other high-income countries.

### Female cases in the outbreak

Of the two female cases in this outbreak, one was infected with the S13 strain reported in 2018, and she had not had contact with other cases or the population of men who have sex with men. Moreover, according to the NESID data, food consumption was the suspected route of infection for this case. However, we did not find evidence of a foodborne outbreak of HAV infection in 2018. Community acquisition could be suspected for this case, but we could not clearly identify the infection route.

### Importance of vaccination

Around two thirds of inpatient cases had an unspecified number of sexual contacts. A previous outbreak investigation reported that such sexual contact was a high-risk behaviour for STIs. (*26*) HIV infection was one risk factor strongly associated with severe complications. (*27*, *28*) Ndumbi et al. (*29*) reported that avoiding faecal–oral exposure during sexual activity and safer sex practices (e.g. use of barrier methods) play important parts in preventing HAV infection and other STIs, including preventing enteric transmission. Additionally, HAV vaccination can protect against faecal–oral transmission and foodborne infection, but we estimated that the seroprevalence of anti-HAV antibodies might be < 10% among those ≤ 60 years of age in Japan. (*30*) Some HAV outbreak investigations have recommended that men who have sex with men should be considered a high-risk population for HAV infection and so should be vaccinated. (*22*, *28*, *31*) Post-exposure prophylaxis is significantly effective in preventing HAV infection. (*32*) However, in two cases, the second dose of the HAV vaccine was received within 10 days before symptom onset. Following close contact with an HAV-positive person, all previously unvaccinated persons should receive the vaccine as soon as possible, preferably within 2 weeks. (*2*) Infection may have occurred in these two cases due to an inadequate amount of time elapsing between vaccination and exposure, thus the vaccine may not have provided adequate protection. Moreover, Japan has not yet implemented universal HAV vaccination, so most residents are not aware of the importance of receiving the vaccine. We recommend that HAV vaccination be given at the appropriate time nationwide.

### Vaccination among men who have sex with men

Most cases in this outbreak had not received the HAV vaccine. Our results showed that cases had only a low awareness of the risk of an HAV outbreak among men who have sex with men and of the importance of vaccination. A past outbreak investigation has described the populations of men who have sex with men as having low rates of HAV vaccination. (*33*) To improve the immunization coverage rate among this population, health-care providers should be made aware of the importance of vaccination, and the population should also be made aware of the availability of vaccination and the importance of asking for the vaccine when they seek routine health care. The United States Centers for Disease Control and Prevention has reported hesitancy to receive HAV vaccination for fear of contracting the disease. (*34*) A study in Australia showed that health-care workers were significantly more aware of vaccination than those in other occupations. (*35*) More frequent contact with health-care providers, especially with a regular physician, could be effective in providing education about HAV infection and promoting timely HAV vaccination among men who have sex with men. (*36*)

### Limitations

This outbreak investigation has some limitations. Around half of the cases were reported from a designated HIV hospital, so some selection bias may have been introduced into the data. Considering that the population is highly vulnerable, we could collect only limited information when tracing sexual contacts. Only half of the inpatient cases among this vulnerable population participated in the interview from which we derived information about risk factors. We could not collect information about the specific SNS tools used by this vulnerable population, such as Tinder or Grindr, as this is sensitive information. Also, our investigation analysed the collection of qualitative data via semistructured interviews to try to understand risk behaviours, and recall bias might be a potential limitation in terms of retrospective data collection. We could not collect details of the clinical course of the illness.

## Conclusions

In 2018, the annual number of reported cases of HAV infection was 131 in Shinjuku, Tokyo, Japan. Of these, 98% were male and 81% were men who have sex with men. We recommend that men who have sex with men, as a population at high risk for HAV infection, should be made more aware of the risk of infection with this STI. This population should also receive HAV vaccination. To improve adherence to safer sex practices, various sources, including local governments, health-care providers and voluntary support groups, can be engaged to widely disseminate information via SNS to improve vital knowledge, attitudes, beliefs and practices regarding the necessity of HAV prevention and vaccination.
